# A Review on Application of Deep Learning Algorithms in External Beam Radiotherapy Automated Treatment Planning

**DOI:** 10.3389/fonc.2020.580919

**Published:** 2020-10-23

**Authors:** Mingqing Wang, Qilin Zhang, Saikit Lam, Jing Cai, Ruijie Yang

**Affiliations:** ^1^Department of Radiation Oncology, Peking University Third Hospital, Beijing, China; ^2^Department of Health Technology and Informatics, The Hong Kong Polytechnic University, Hong Kong, China

**Keywords:** artificial intelligence, machine learning, deep learning, automated learning, radiotherapy

## Abstract

Treatment planning plays an important role in the process of radiotherapy (RT). The quality of the treatment plan directly and significantly affects patient treatment outcomes. In the past decades, technological advances in computer and software have promoted the development of RT treatment planning systems with sophisticated dose calculation and optimization algorithms. Treatment planners now have greater flexibility in designing highly complex RT treatment plans in order to mitigate the damage to healthy tissues better while maximizing radiation dose to tumor targets. Nevertheless, treatment planning is still largely a time-inefficient and labor-intensive process in current clinical practice. Artificial intelligence, including machine learning (ML) and deep learning (DL), has been recently used to automate RT treatment planning and has gained enormous attention in the RT community due to its great promises in improving treatment planning quality and efficiency. In this article, we reviewed the historical advancement, strengths, and weaknesses of various DL-based automated RT treatment planning techniques. We have also discussed the challenges, issues, and potential research directions of DL-based automated RT treatment planning techniques.

## Introduction

As one of the cancer treatment modalities, radiotherapy plays an important role in the treatment of numerous types of malignant tumors. Treatment planning is an important process of radiotherapy. Advanced algorithms are used to calculate dose deposition and optimize a treatment plan by taking into account the planning computer tomography (CT) images and a range of dosimetric objectives. Radiation beam parameters, including aperture shapes at each gantry angle and dose deposition for each aperture, are also determined during the treatment planning process. The beam parameters are subsequently transferred to radiotherapy machines to enable radiation delivery so that the prescribed dose distribution can be delivered as planned to achieve satisfactory tumor control while preserving normal tissue function (1).

The current practice of treatment planning is largely a manual process, which is time-consuming and labor-intensive, typically taking hours, or days to complete one case. The plan optimization parameters need to be manually adjusted and determined by planners. Hence, the plan quality heavily depends upon the planner's experience. It is a trial-and-error process through multiple iterations to balance the trade-off between tumor control and normal organs complications, which requires plenty of human interactions. The iterative nature of manual treatment planning makes it a tedious process. It requires experienced planners, particularly for highly complex plans, leading to prodigious human efforts, expertise, and time commitment. Consequently, the quality of a plan created by different planners can be largely inconsistent and limited by practical considerations, such as the proficiency of planners and inadequate efforts made to design an “optimal” treatment plan, even though the plan is clinically acceptable (2).

Automated treatment planning (ATP), which is developed to overcome the challenges mentioned above of manual treatment planning, is capable of generating treatment plans that are of sufficiently high quality and high consistency in a time-saving manner (3). Automated treatment planning has evolved from simple template-based, atlas-based automation execution to machine learning, and deep learning-based DVHs or dose distribution prediction, to direct plan parameters generation. The current ATP solutions include automated rule implementation and reasoning (ARIR), and modeling of prior knowledge-based approaches. AutoPlanning from Philips Pinnacle treatment planning system (TPS) is an example of ARIR based methods (4–6), which firstly constructs a template including many kinds of clinical protocols, such as target and organ-at-risk (OAR) prescriptions. The TPS then begins plan optimization just like a planner and finally obtains a solution based on the selected clinical protocol, to avoid excessive radiation dose being deposited to healthy tissue while maximizing tumor coverage. In the optimization process of AutoPlanning, some supporting structures are created to improve the OAR sparing. RapidPlan, a built-in automated knowledge-based planning (KBP) module in Varian (Varian Medical System) Eclipse TPS, is an example of modeling of prior knowledge. It utilizes a large amount of historical patients' planning data to fit a principal component analysis-based regression model for predicting a new patient's dose-volume histograms (DVHs), which are subsequently used to guide the generation of a new treatment plan (7–16).

No spatial dose distribution information was considered in the DVH-based prediction methods. Compared with the DVH-based prediction algorithms, algorithms for directly predicting 3D dose distribution have significant advantages in a way that it could provide a reference for clinical adjustment for distribution of isodose lines. To solve the problem of lack of spatial dose distribution, and the degeneracy issue of KBP DVHs estimations, recent developments in ATP have focused on voxel-level dose prediction (17, 18). With the rapid advancement of machine learning and deep learning (19, 20), the accuracy of these prediction methods was increased substantially (17, 21). Artificial intelligence (AI) includes all approaches to imitate human intelligence by machines. ML is a branch of AI, and deep learning is a form of ML. The idea of DL was inspired by the structure and function of brain (19, 20). The first DL method was an artificial neural network algorithm, and the neural network was composed of numerous nodes and connection weight, analog to the neurons and connection of neurons in the brain. A variety of DL techniques has been explored and investigated over the past decade. Initially, there were only input and output layers in the first generation, called single-layer neural network. Multi-layer neural networks have later evolved with hidden layers added into the neural networks. The multi-layer neural network with only one hidden layer was called shallow neural network, while those with two or more hidden layers were called deep neural networks—the origin of the nomenclature of deep learning methods. From artificial neural network (ANN), convolutional neural network (CNN), to generative adversarial network (GAN), the emergence of different network structures has led to different DL methods. The biggest difference of the three kinds of neural networks is their structure. ANN is a mathematical model that simulates the processing information of neurons, and it is usually composed of three parts: input layer, hidden layer, and output layer. However, for a very deep neural network (a network with a large number of hidden layers), the Vanishing gradient or exploding gradient problems arise when propagating backward. CNN is the first true multi-layer structure learning algorithm, which uses spatial relative relationships to reduce the number of parameters to improve training performance. On the basis of the original multi-layer neural network, a feature learning part is added, which imitates the human brain's classification of signal processing. Therefore, compared with ANN, the specific operation of CNN is to add a partially connected convolutional layer and a dimensionality reduction layer in front of the original fully connected layer, which are used for feature extraction from different dimensions automatically. The GAN consists of two parts: one is called generator, and the other is discriminator. The generator tries to generate data that is close to real, and the discriminator tries to distinguish between real data and data generated by the generating network. The generator network uses the discriminator as a loss function and updates its parameters to generate more realistic looking data. On the other hand, the discriminator network updates its parameters in order to better identify fake data from real data. So its performance has become better. This cat and mouse game continues until the system reaches the so-called “balance.” After reaching the balance, the data created by the generator looks realistic enough, so all the discriminator can do is random guessing.

The DL methods can be divided into three classes: supervised DL, unsupervised DL, and reinforcement learning. The main differences between the three classes are the input terms used in different deep learning network architectures. The training dataset of supervised deep learning algorithms consists of input and corresponding output data, while for the unsupervised learning methods; only input data is included in the dataset without labeled output data. Compared with these two techniques, reinforcement learning requires different types of data: the input, partial corresponding output, and grade for output. By training these datasets, a deep learning model can be obtained for predicting the output results.

In recent years, a number of deep learning (DL)-based ATP techniques have been proposed using various DL neural networks (18–33). Several review articles on AI in radiation oncology (34–36), and radiotherapy treatment planning (37–39), have been published, which demonstrated the interests on AI and the significance of ATP, summarization of the achievements and challenges, as well as insightful discussion on future studies. No comprehensive review specifically focused on deep learning-based automated radiotherapy planning was published. In this review, we will summarize the historical advancement and current status of automated radiotherapy planning using deep learning, including the advantages, and limitations of various DL-based ATP techniques. The challenges, practical issues, and potential research directions of DL techniques in ATP are also discussed.

## Deep Learning for Automated Treatment Planning

Conventional radiotherapy treatment planning usually consists of inverse optimization with trial and error (40). Correspondingly, the automated treatment planning can be summarized into three steps: automated beam orientation selection, automated dose map prediction, and automated fluence map and delivery parameters generation. A detailed introduction is carried out in the following text.

## Automated Beam Orientation Selection

In 2020, Barkousaraie et al. (41) proposed a supervised DL algorithm mimicking the column generation (CG) method for beam direction optimization, saving time to seconds, and increasing the possibility of clinical use. In the algorithm, 57 prostate cancer patients were utilized for training and validation, 13 patients for testing. The result showed that the differences between plans generated by CG and by DL algorithm in the dose coverage of PTV were about 0.2%. The mean dose differences received by organs at risk were under 6%. Additionally, a reinforcement learning method was developed for improvement of the deep learning algorithm (42). It can be summarized into two steps: Firstly, the possible next beam distribution is predicted based on patient anatomy, by training a supervised deep neural network; and, to find better solutions, a guided Monte Carlo tree search method, combined with the network, is utilized for decision. The result showed that the differences between plans generated by CG and by reinforcement learning algorithm in the dose coverage of PTV were similar. The mean dose differences received by organs at risk could be reduced except for bladder, which had a slight increase of 1%. These two deep learning methods have been proved more accurate than the state-of-art CG method. It is a promising direction for automated beam orientation selection.

## Automated Dose Map Prediction

In 2016, Shiraishi et al. (18) developed a three-dimensional (3D) dose distribution prediction method based on an ANN using geometric and planning parameters of historical patients. The prediction error for all voxels was <8% for tested prostate cases. The three-dimensional (3D) dose distribution prediction is an improvement to one-dimensional (1D) DVHs prediction, which makes voxel-by-voxel dose optimization and knowledge-based isodose manipulation possible. Since then, significant efforts have been made by researchers, and the use of deep learning in dose prediction has been extensively explored. Different architectures of CNNs have been utilized for 3D dose distribution prediction. Campbell et al. (21) developed an ANN 3D dose distribution prediction model for pancreatic stereotactic body radiation therapy (SBRT) delivered using 2–4 coplanar arcs. The network was trained using forty-three clinical plans with plan parameters and voxel-based geometric parameters. Plan parameters included the photon beam energy and PTV volume. Geometric parameters included the voxel's distance to the PTV, distance to an OAR, and the number of arcs directly irradiating the voxel. The predicted mean dose errors were <5%. Excellent model performance was demonstrated for the volume receiving dose above 25 Gy, but much larger prediction errors were seen at the lower dose region. The prediction accuracy was significantly improved when each physician's treatment protocols, and treatment approach, were taken into account by training their dedicated models separately. Kajikawa et al. (29) predicted the dosimetric eligibility of prostate cancer patients treated with IMRT using a convolutional neural network called Alex-Net. The Alex-Net was pre-trained with a big and open dataset called Image-Net, and then modified with a new CT dataset. Unlike other methods, the prediction result is not a dose distribution but two categories that meet all dose constraints category or do not meet all dose constraints category. The prediction errors of the model with the planning CT image dataset without and with the structure label were 56.7 ± 9.7% and 70.0 ± 11.3%, respectively. Compared with previous methods, such as the ANN method, the 2D, and modified 3D U-Net methods, this method was worse in terms of prediction accuracy. However, it opened a new direction for ATP using transfer learning.

Nguyen et al. (22) proposed a modified 2D U-Net architecture for dose distribution prediction using labeled targets and OARs of 88 coplanar prostate intensity-modulated radiation therapy (IMRT) plans as input. Each patient was planned with a similar beam configuration and prescription protocol. The planning CT images were input into the full convolutional networks directly, no handcrafted feature extractions or selections were needed. The predictions were based on more detailed geometric and anatomical information than parametric or principal component analysis approaches. The predicted average absolute dose difference was around 2% of the prescription dose in PTV and under 5% in OARs. The average dice coefficient of the predicted isodose volumes and the actual isodose volumes was 0.91. The 2D U-Net algorithm is different from the ANN approach, in which handcrafted feature extractions or selections were used. Different input features may lead to different model performance, which is often considered a lack of consistency among different users, time-consuming, and labor-intensive. The 2D-U-net provided better predictive performance compared with previous ANN method (18). However, the most significant barrier of this 2D U-Net method is that it predicts the 3D dose distribution on a slice-by-slice basis, rather than a true 3D volumetric prediction. This kind of prediction may cause uncertainties, especially in the edges of the PTVs, and the whole training process can be tedious.

Nguyen et al. (23) further proposed a hierarchically densely connected U-Net (HD U-Net) for 3D dose distribution prediction for head and neck cancer patients treated with volumetric-modulated arc therapy (VMAT) based on the combination of U-Net (24) and Dense-Net (25). U-Net was initially proposed as a deep learning approach for semantic segmentation (24). The previous maps are concatenated to the following layers in the CNN of DenseNet (25). A 3D densely connected U-Net combining the advantages of U-Net and Dense-Net was developed with a reduced random-access memory (RAM) requirement. The convolution layers are connected densely along each hierarchy, but not connected between hierarchies of the U-Net in the upsampling operation. The issue of vanishing gradient was solved by connecting the convolutional maps densely to promote feature propagation and reuse, and the number of trainable parameters needed was also reduced. HD U-Net is capable of predicting the dose distribution accurately from the PTV and OAR contours. The predicted organ-at-risk maximum dose was within 6.3% and mean dose within 5.1% of the prescription dose. Kearney et al. (28) proposed a fully convolutional neural network (Dose-Net) for 3D dose prediction of prostate cancer patients treated with non-coplanar SBRT. CT images, structures, and dose prescriptions were used as input in the 3D fully-convolutional multi-channel Dose-Net. Compared with U-net, Dose-Net reduced network redundancy due to the use of residual blocks. However, fully connected layers tend to generalize poorly for high dimensional data. Considering both using bladder patients' data as training data, a comparison between Dose-Net and modified U-Net has been conducted. According to the results of the two articles, the absolute differences of Dose-Net and modified U-Net in PTV, bladder, rectum in [max, mean] dose are [−2.5, 0.2%] vs. [1.80, 1.03%], [9.9, 2.5%] vs. [1.94, 4.22%], [8.3, 1.6%] vs. [1.26, 1.62%]. It could be concluded that Dose-Net shows more robust performance than modified U-Net, because Dose-Net has lower mean dose differences than modified U-Net. Chen et al. (30) used CNN called Res-Net for predicting optimal dose distributions of nasopharynx carcinoma (NPC) IMRT plans with the planning images and segmented anatomy as input. They found that the prediction accuracy of out-of-field dose distributions was improved by adding radiation beam geometry as input. Liu et al. (31) proposed a Res-Net for predicting dose distributions of NPC patients treated with Tomotherapy, with the contoured PTVs and OARs, dose volumes, and CT images as input. The predicted mean absolute dose differences are within 2.0 and 4.2% for PTVs and OARs, respectively. Fan et al. (32) also used Res-Net for NPC dose prediction with the CT images containing OARs and PTVs being set as input terms. The differences between the predicted dose and the clinical dose were not significant except for structures of brainstem and lens at both sides. They further generated a deliverable plan based on predicted dose distribution. No significant differences were found between the dose distribution of the generated plan and the predicted dose distribution except the difference of 0.5% for PTV70.4.

Also, based on a combination of U-Net and Dense-Net, Barragán-Montero et al. (26) developed a more general model considering variable beam setups in addition to patient anatomy. The beam setups are represented by an approximate cumulative dose distribution from the involved beams. The model considering beam configuration and patient anatomy outperformed the model considering only patient anatomy, especially in the medium and low dose regions for lung IMRT plans in predicting spatial dose distribution with varying beam arrangement. This approach makes it unnecessary to train different models for different beam setups. This is especially important for IMRT, including non-coplanar treatment techniques for lung, brain, or liver, among others. For these sites, the spatial relationships of the tumor with organs at risk vary among different patients and the beam setups also vary much more than for prostate. Zhou et al. (27) also improved a 3D U-Res-Net model performance to predict 3D dose distribution for postoperative rectal cancer patients treated with IMRT considering beam configurations input.

Apart from various CNN models, GAN models have also been utilized for 3D dose distribution prediction. Mahmood et al. (33) recast the dose prediction problem as an image colorization problem solved with two neural networks: a generator performing a task and a discriminator evaluating the performance of the generator. For treatment planning, the generator plays the role of a planner, while the discriminator plays the role of a radiation oncologist who evaluates the plan designed by the planner. Both networks were trained simultaneously on historical data, with effective replication and aggregation of the combined knowledge and experience gained during the iterative manual process used to design clinically acceptable plans. They used contoured CT images and clinically acceptable dose distribution from the treatment plans of past oropharyngeal cancer patients to train a GAN. It was found that the GAN algorithm outperformed a query-based approach, a principal component analysis-based method, a random forest, and a CNN method, and even outperformed the clinical plans on clinical criteria satisfaction. A major drawback of most existing prediction methods is their reliance on low dimensional manually made features in patient geometry to predict dose distributions. GAN eschews the classical paradigm of predicting low-dimensional representations, or engineering features, by training a generic neural network to learn desirable dose distributions (43). The capability of GAN in generating truly independent data, and producing an optimal treatment plan need further investigation (44).

## Automated Fluence Map Generation and Delivery Parameters Generation

After the predicted dose distribution is obtained, the next step is to convert the dose distribution into an executable plan. Conventionally, there are two main methods to do this: dose mimicking and inverse optimization (32, 33, 45). The dose mimicking algorithm penalizes dose discrepancies between post-mimicking dose and input dose by minimizing the L2 norm loss, where the post-mimicking dose should satisfy all the constraints and objectives (32, 45). Dose mimicking has been incorporated into commercialized RayStation TPS from RaySearch Laboratories.

In the era of artificial intelligence, deep learning has been utilized for fluence map generation, with different neural networks architectures being tested (46, 47). In 2019, Lee et al. (46) proposed a modified U-Net algorithm to train with 240 prostate IMRT plans and 45 plans for test (46). The result showed that the final synthetic plans had worse homogeneity index of the target, and had nearly the same performance in conformity index of the target. In 2020, Li et al. (47) proposed a Dense-Res Hybrid Network (DRHN) algorithm to train with 106 prostate IMRT plans and 14 plans for test. The result showed that the final synthetic plans were compatible with the KBP plans and clinical plans, with less time consuming. These two methods prove the effectiveness of deep learning in predicting fluence map.

## Discussion

### From Machine Learning to ANN, to CNN

Various types of ATP approaches have been investigated in the past few years. The machine learning-based approaches are the most extensively investigated and have significantly improved the DVHs prediction accuracy. These approaches require handcrafting features for DVHs prediction. Many efforts have been made in selecting the handcrafted features, such as distance-to-target histograms, the geometry of PTV and OARs, overlapping volume histograms, etc. (8, 48–52). It is hard to know which features impact the prediction most and what other features could improve the performance of the model significantly. ANN was investigated to learn the more complex relationships between the handcrafted features and the predicted DVHs or dose distribution. However, the inherent information present in those data is still limited. Compared with ANN, CNN improved the voxel-based dose distribution prediction, due to its ability to extract local and global features from the patients' CT images in higher dimensions.

### From DVH Prediction to Dose Distribution Prediction, From the 2D Model to 3D Model

Considering the limitations of DVHs prediction, more recent studies focus on spatial dose distribution prediction, as shown in Table 1. The prediction models also evolve from the 2D model to the 3D model. The most investigated DL-based ATP approaches have realized that CT images, structures, and dose distribution maps are taken as input terms. When putting new patients' CT images and structure labels into the constructed model, the predicted dose distribution could be obtained and exported as the output, which is then further converted to yield the ultimate deliverable plans. Kajikawa et al. (55) found that the dose predicted with the 3D CNN model was superior or comparable with the dose distribution generated by RapidPlan TM for prostate cancer IMRT plans using only contours in planning CT. Ma et al. (56) incorporated the dose distribution from a PTV-only plan, in addition to the patient's structures contour data from planning CT in their deep CNN-based dose prediction model. The prediction results were better than the contours-based method. CT value also influences the dose distribution in addition to the PTV and OARs contours for a given beam configuration. The significance of adding the CT value as additional input information into the prediction model needs to be further evaluated in future studies. The architectures Alex-Net, VGG-Net, U-Net, V-Net, and Res-Net belong to the category of CNN and are all investigated in detail. CNN is the most frequently used technique for 3D dose distribution predictions.

**Table 1:** Selected studies on deep learning-based automated radiotherapy planning.

**Reference**	**Year**	**Network**	**Training sets**	**Test sets/NO**.	**Input**	**Output**	**Results**	**Research Highlight**
Shiraishi et al. (18)	2016	ANN	23 prostate and 43 SRS/SRT VMAT plans. Twelve training and 11 validation for prostate, and 23 training and 20 validation for SRS/SRT	No	Manually determined geometric and plan parameters	3D dose	Prediction errors <10% and 8% for prostate and SRS/SRT cases, respectively	Knowledge-based 3D dose predictions, rather than previous 1D DVH prediction
Campbell et al. (21)	2017	ANN	43 pancreatic Arc-based SBRT patients. Nineteen training and 10 validation for Group A, 9 and 5 for Group B, respectively	No	Plan parameters and voxel-based geometric parameters	3D dose	Mean dose error <5%	Prediction accuracy substantially improved when each physician's treatment approach was taken into account by training their own dedicated models
Nguyen et al. (22)	2017	Modified 2D-Unet	80 prostate IMRT patients, 10-fold cross-validation	8	labeled targets and OARs	3D dose	Prediction errors around 2% in PTVs and under 5% of the prescription dose in OARs, isodose volumes average dice coefficient of 0.91	Unet for 3D dose prediction
Nguyen et al. (23)	2019	3D HD U-Net	100 H&N VMAT patients, 5-fold cross validation	20	Labeled targets and OARs, prescription doses	3D Dose	OARs dose difference :maximum error within 6.3% and mean error within 5.1%	Outperforming the Standard U-net and Dense-Net in both prediction accuracy and efficiency
Barragán-Montero et al. (26)	2019	3D HD U-Net	100 lung IMRT patients, training, and validation	29	Labeled targets and OARs, beam setup information	3D Dose	Prediction accuracy improved substantially in low and medium dose regions and slightly in high dose regions	Prediction accuracy improved by considering beam setup information
Zhou et al. (27)	2020	3D U-Res-Net	100 rectal cancer postoperative IMRT patients	22	Labeled targets and OARs, beam setup information	3D Dose	Mean absolute prediction errors 3.92 ± 4.16%,clearly outperforming 3D U-Res-Net_O and slightly superior to 3D U-Net	Prediction accuracy improved by considering beam setup information
Kearney et al. (28)	2018	FCNN Dose-Net	126 prostate non-coplanar SBRT Cyberknife patients, 106 training, 20 validation	25	Labeled targets and OARs, dose prescription	3D Dose	A superior alternative to U-Net and fully connected network	Utilizes a 3 phase learning protocol to achieve convergence and improve generalization
Kajikawa et al. (29)	2018	Alex-Net CNN	60 prostate IMRT patients, five-fold cross-validation	No	CT images, with/without labeled structures	3D dose	Prediction accuracies 56.7 ± 9.7% and 70.0 ± 11.3%, respectively	Pre-trained on Image-Net database, the model with structure labels focused on areas related to dose constraints improved prediction accuracy
Chen et al. (30)	2018	Transfer learning ResNet	70 early-stage NPC IMRTpatients	10	Labeled targets and OARs, with/without beam setup information	2D dose map	Out-of-field dose distributions prediction error 4.7 ± 6.1%vs. 5.5 ±7.9%, input with/without beam setup information	Input information from beam geometry improved the out-of-field dose distributions prediction accuracy
Liu et al. (31)	2019	U-ResNet-D	170 NPCTomotherapy patients, 136 training, 34 validation	20	Labeled targets and OARs,3D dose	3D Dose	Mean absolute dose differences for PTVs and OARs are within 2.0 and 4.2%, respectively	U-ResNet-D for Tomotherapy 3D dose prediction
Fan et al. (32)	2019	ResNet	270 H&N IMRT patients, 195 training, 25 validation	50	Labeled targets and OARs	3D Dose	Predicted differences not statistically significant for clinical indices of all targets and OARs except the difference of 0.5% for PTV70.4	Automatic plan generation based on predicted 3D dose distribution
Mahmood et al. (33)	2018	GAN	130 oropharyngeal IMRT patients	87	Labeled targets and OARs, dose maps	3D dose	Outperformed a query-based, a PCA-based method, a random forest, and a CNN method, on clinical criteria satisfaction	Recast the dose prediction problem as an image colorization problem, solve the problem using a GAN by mimicking the iterative process between the planner and oncologist
Appenzoller et al. (8)	2019	3D CNN	80 prostate IMRT patients	15	Labeled targets and OARs	3D dose	Prediction error: 1.10 ± 0.64%, 2.50 ± 1.17%, 2.04 ± 1.40, and 2.08 ± 1.99% for D_2_,D_98_ in PTV-1 and V_65_ in rectum and V_65_ in bladder	3D CNN was superior to or comparable with RapidPlan^TM^
Krayenbuehl et al. (50)	2019	CNN	60 prostate VMAT patients	10	Labeled targets and OARs, the dose distribution from a PTV-only plan	3D dose	Mean SARs for the PTV, bladder, and rectum 0.007 ± 0.003, 0.035 ± 0.032, and 0.067 ± 0.037, respectively	Prediction results better than the contours-based method
Shin et al. (53)	2019	DNN	240 prostate IMRT plans	45	Labeled targets and OARs, dose distributions	Fluence-maps	Generated plan qualities comparable with the corresponding clinical plans	Generate beam fluence—maps directly from the organ contours and dose distributions without inverse planning
Wieser et al. (54)	2020	DNN,DRL-based VTPN	10 prostate IMRT patients	64	IMRT plans	IMRT plans	Spontaneously learn how to adjust treatment planning parameters, high-quality treatment plans generated	The first artificial intelligence system to model the behaviors of human planners in treatment planning

*ANN, artificial neural network; SRS, stereotactic radiosurgery; SRT, stereotactic radiotherapy; VMAT, volumetric-modulated arc therapy; 3D, three dimensional; 1D, one dimensional; DVH, dose-volume histogram; SBRT, stereotactic body radiation therapy; 2D, two dimensional; IMRT, intensity-modulated radiation therapy; OARs, organs at risk; PTV, planning target volume; HD U-Net, Hierarchically densely connected U-Net; H&N, head and neck; U-ResNet-D, model looks like U-net, but uses ResNet to do down-sampling and deconvolution to perform up-sampling; FCNN, fully convolutional neural network; NPC, nasopharynx cancer; GAN, generative adversarial network; PCA, principal component analysis; MAE, mean absolute errors; SARs, sum of absolute residuals; DNN, deep-neural-network; DRL, deep reinforcement learning; VTPN, virtual treatment planner network*.

### Size of Data Sets, Model Over-Fitting, and Generalization Performance

One issue of the current DL-based ATP approach is the severe lack of high-quality and publicly available big datasets. Most of the datasets reported in this review article involved <300 patients, which is not an adequate sample size under the context of big data. In addition, model over-fitting caused by data imbalance is also an important issue in ATP generation. The plan quality and prescription variation in the training and test dataset influences the model prediction error. It has been found that the dose prediction accuracy was improved by training the plans from two different radiation oncologists separately (21).

Also, the systematic training, validation, and testing require a sufficiently large, high-quality database. The training data and validation data should be separated from the test data for a rigorous model training and testing process. Without enough data for model training and testing, over-fitting tends to occur due to an overly complex model produced from a limited data set. The more complex model was shown to perform inferiorly to the simpler ones for dose prediction in the previous study (57). Therefore, high-quality databases with optimal plans should be established. Improvement of manual planning experience of existing personnel, classic optimization algorithm, multiple institution cooperation, and standardized protocols are benefiting for the DL-based ATP.

The model over-fitting influences the model generalization performance. One method to address limited data size is using transfer learning. Image-Net was commonly used for pre-training deep learning networks for medical image processing (53). Another method for addressing the issue of limited data set is data augmentation.

### Clinical Scenarios Complexity

More clinical conditions, such as different tumor sites and delivery techniques, also need to be further investigated for DL-based ATP. For fluence map generation, the two articles give two different deep neural network architectures for fluence map generation with prostate plans, other plans in different tumor sites need to be tested to find the optimal deep neural network architecture. In the meanwhile, when a new patient's fluence map is predicted with deep learning, leaf motion calculation and multi-leaf collimator (MLC) leaf sequence are still required to be determined to obtain the final machine parameters. Prostate cancer patients have more consistent PTV sizes and spatially neighboring anatomy than lung cancer or head and neck cancer patients. Coplanar IMRT is different from non-coplanar VMAT in terms of the beam configurations. The diverse clinical scenarios determine the poor data uniformity, and the data with good data consistency is scarce. It requires various networks and training techniques of transfer learning and data augmentation to mitigate over-fitting. Also, the effect of the weight decay, learning rate, momentum strength, and other hyper-parameters, and new architectures for more intelligent networks should be tested (58). More types of data, including multimodality images, and genomic data, can be integrated into the deep learning-based automated planning.

### Direct Prediction of Machine Parameters

For beam orientation selection with deep learning, the current research focuses on IMRT plans solution, however, partial arc volume modulated treatment (VMAT) plans have not been solved. It is also very important to determine the start and end angles of partial arc VMAT plans. In addition, the beam orientation selection and other machine parameters determination with deep learning for non-coplanar IMRT and VMAT plans may be another trend in the future. Most recent studies of ATP using DL focused on dose distribution predictions. After dose distribution prediction, the predicted dose distribution was converted into executable plans using inverse optimization or dose mimicking (32, 33, 45). Direct machine parameter prediction could become another potential research area, which is promising in that the plan optimality, deliverability can be considered and balanced in addition to the prediction accuracy. It has been observed that ATP has systematically increased the modulation factor (37). As deep learning shows increasing promise for automated image segmentation, direct deliverable plan generation by using CT images without contoured structures, without dose distribution is possible for automatic dose prediction. Lee et al. (46) investigated a deep-neural-network to generate beam fluence maps directly from the structure contours and 3D dose distributions without inverse planning. The generated plan qualities were comparable to the corresponding clinical plans.

### Reinforcement Learning

Reinforcement learning has been used successfully in the board game Go (59). Shen et al. (60) investigated a deep reinforcement learning-based network to model the behaviors of human planners. In the treatment planning process, a series of actions depend on the balance of targets and OARs dose distribution. This feature makes that reinforcement learning is suited for treatment planning.

Extensive research efforts have continuously been made to develop a wide range of innovative structures of deep neural networks in recent years, such as graph neural networks (GNN), graph convolutional networks (GCN), graph reinforcement learning (GRL), graph auto-encoders (GAE), and graph regression neural networks (GRNN) (59, 61). This diversity of neural network architectures has unarguably facilitated the development of DL-based 3D dose prediction strategies. Nevertheless, several inherent shortcomings of the neural network algorithm remain to be addressed, such as black box problem, time-consuming, labor-intensive, large amount of data required, and so on.

### Model Prediction Accuracy and Clinical Non-inferiority

Another issue of deep learning-based ATP studies is the lack of consensus on determining an “optimal” while clinically acceptable treatment plan in an objective manner. To evaluate the ATP model prediction accuracy, various quality metrics were usually used to compare with the existing manual clinical plans. Dose difference and statistical significance of points in the volume of interest in ATP plans and clinical plans were commonly used. Visualization of DVH differences with clinical significance was also used for structures of interest, which highlights outlier instances better than summary statistics. Besides, voxel-based mean absolute error, global or structure-based three-dimensional gamma analysis, was also used for evaluation and loss function. One ideal solution of evaluating the model prediction accuracy and plan optimality is to quantitatively score treatment plans integrating clinically significant quality metrics, such as homogeneity, conformity, or the entire dose distribution itself, in addition to the above mentioned. The indices for evaluating the prediction accuracy and plan optimality of the ATP model should be established in addition to an open, high-quality database to compare the performance of the different models.

Besides the evaluation of each quality metric, an alternative approach is a blinded side-by-side comparison of automated and manual plans. If the automated plans are indistinguishable from the manual plans, then the ATP system can be used under the supervision of the planners and radiation oncologists. It should be noticed that a “problem” with DL is that it does not provide any insight on plan optimality, and also Pareto-optimality is not ensured. To mitigate this, a QA should be present.

### Legal and Ethical Consideration

The issues of patient safety, legal and ethical responsibilities need to be considered before deep learning-based ATP is put into clinical practice. Currently, deep learning algorithms are often regarded as a “black box,” and the internal working mechanism is still largely unknown, thus highlighting the seriousness of this problem. Consequently, there is a pressing demand for a thorough, comprehensive, and rigorous quality assurance program for DL-based ATP strategies and software to maintain a sufficiently high consistency of the generated plans with full compliance to a set of safety standards. Scoring software and independent third-party evaluation software could potentially serve as solutions to address the issues stemming from automatic planning algorithms.

### Preclinical Validation

The automatic planning algorithms must be validated before being used in the clinic for safety and quality. A large size data set is needed for the preclinical validation of deep learning-based ATP. Multi-center collaborations should be encouraged to cumulate adequate data for the ATP system development and validation, and generalization. Multi-institutional prospective studies with detailed planning guidelines and protocol compliance are helpful in obtaining high-quality data for deep learning-based ATP development and validation. Open platforms and software packages can be used for the development and validation of deep learning-based ATP (54, 62). New regulation and supervision of data should be available to encourage the DL-based ATP development and guarantee the data security and proprietary intellectual property.

### Quality Assurance

As ATP systems improved the planning efficiency with comparable or even better plan quality, systematic, and comprehensive quality assurance program should be established and implemented after preclinical validation. The automatically generated plans may not be “optimal.” The quality assurance and monitoring of ATP should be investigated with top priority and extensively. Even if the ATP system has proved its performance for tested cases, no one can assure their performance for the new cases. Whenever new cases with different geometric and dosimetric characteristics are put into the ATP system, the new generated ATP plans should be reviewed carefully. The clinical ATP process must be overseen closely and continuously by physicist and radiation oncologist.

### Model Adaptability

The ATP system should be adaptable to new emerging trade-offs or knowledge. The criteria and evaluation of plan optimality evolve with the advancement of new diagnostic and therapeutic technology. Examples include but are not limited to the change of prescription dose and constraint due to the integration and development of chemotherapy, target therapy, and immunology; the evolution of targets and OARs contouring due to the application of new functional molecular imaging modality. The ATP system needs to adapt to these changes in the context and judgment criteria of plan optimality.

### Summary

In recent years, various types of ATP solutions have been proposed and investigated, and the results demonstrated measurable improvement in plan quality and planning efficiency. Deep learning-based ATP is a rapidly evolving field. It holds great promises to be a highly useful tool for automatic plan generation, plan quality evaluation and quality assurance, individualization of dose prescription, and adaptive radiotherapy, etc. Further studies are needed to address the remaining issues. Cautions should be taken with regard to its limitations before it is implemented for routine clinical use.

## Author Contributions

MW and RY wrote the manuscript. QZ helped for article selection and data analysis. SL and JC helped for technical review of the manuscript for deep learning and clinical aspects. RY performed a technical review of the manuscript on deep learning and clinical aspects, and also for manuscript redaction. All authors contributed to the article and approved the submitted version.

## Conflict of Interest

The authors declare that the research was conducted in the absence of any commercial or financial relationships that could be construed as a potential conflict of interest.
